# Extinction-resistant attention to long-term conditioned threat is indexed by selective visuocortical alpha suppression in humans

**DOI:** 10.1038/s41598-019-52315-1

**Published:** 2019-11-01

**Authors:** Christian Panitz, Andreas Keil, Erik M. Mueller

**Affiliations:** 1 University of Marburg, Department of Psychology, Gutenbergstr. 18, 35032 Marburg, Germany; 2 University of Florida, Center for the Study of Emotion and Attention, 3063 Longleaf Road, Gainesville, FL 32608 USA

**Keywords:** Visual system, Classical conditioning, Fear conditioning, Emotion, Long-term memory

## Abstract

Previous electrophysiological studies in humans have shown rapid modulations of visual attention after conditioned threat vs. safety cues (<500 ms post-stimulus), but it is unknown whether this attentional prioritization is sustained throughout later time windows and whether it is robust to extinction. To investigate sustained visual attention, we assessed visuocortical alpha suppression in response to conditioned and extinguished threat. We reanalysed data from *N* = 87 male participants that had shown successful long-term threat conditioning and extinction in self reports and physiological measures in a two-day conditioning paradigm. The current EEG time-frequency analyses on recall test data on Day 2 revealed that previously threat-conditioned vs. safety cues evoked stronger occipital alpha power suppression from 600 to 1200 ms. Notably, this suppression was resistant to previous extinction. The present study showed for the first time that threat conditioning enhances sustained modulation of visuocortical attention to threat in the long term. Long-term stability and extinction resistance of alpha suppression suggest a crucial role of visuocortical attention mechanisms in the maintenance of learned fears.

## Introduction

Learning to predict threat and safety based on environmental cues is fundamental for adaptive behaviour. A well-established paradigm for elucidating mechanisms of associative cue-outcome learning is classical threat conditioning (also fear/aversive conditioning) where conditioned stimuli are threat cues (CS+) when they signal an upcoming aversive event (unconditioned stimulus; US) and safety cues (CS−) when they signal the absence of an aversive event^1–3^. The acquired conditioned defensive response to the CS+ is expected to diminish again upon extinction training, i.e., repeated presentation of CS+ without US^4^.

Importantly, successful short-term learning – i.e., acquisition and extinction of a conditioned response within an experimental session – is a prerequisite for long-term learning^4^. However, memory traces additionally need to be consolidated, retained, and recalled in future situations if learning experiences are to shape adaptive behaviour in the long run. Such long-term learning effects are indexed by the successful recall of previously acquired and extinguished contingencies in a delayed test session^4^. Importantly, acquisition and extinction learning form separate memory traces and individuals may recall previously acquired threat memories without recalling extinction memories. In other words, they may show long-term extinction resistance, which is assumed to be a key process in the maintenance of fears^5^. In this case, despite the absence of aversive events, individuals keep showing robust conditioned threat responses over time.

Conditioned threat responses have different functions and manifest at various levels of central and autonomic physiology. Among these, increased attentive processing promotes capture of threat-relevant information, improving chances of successful defensive responding^6^. Supporting this notion, studies using visual evoked brain potentials in humans have found selectively heightened visual attention when viewing threat cues^7–12^. However, because visual evoked potentials to threat generally reflect early brain activity (i.e., <500 ms)^13^, it is unknown whether enhanced visual attention is sustained throughout later visual processing stages. Moreover, it is unclear, if heightened attention displays long-term resistance to extinction (i.e., occurs despite earlier extinction training). These are clinically relevant questions given that enduring prioritization of threat processing may interfere with fear reduction through extinction and exposure therapy. The present study addressed these questions using a robust electrophysiological marker of sustained visual attention, suppression of visuocortical oscillatory activity in the alpha range.

Alpha oscillations (e.g., 8–12 Hz)^14,15^ are thought to index inhibitory processes in neural populations^16,17^. Visually evoked posterior alpha suppression has been associated with increased excitability of early visual areas in the occipital cortex^18–21^ in response to increased attentional demands^22–25^. Moreover, posterior alpha power suppression has been found stronger in response to aversive vs. neutral/appetitive pictures^14,26,27^ (but also see^28^), taken to reflect prioritized visual processing of threat information^6^.

In the present study, we reanalysed data from *N* = 87 participants that had completed a two-day differential threat conditioning paradigm with threat acquisition and extinction on one day and a critical recall test one day later^29^. We previously reported successful long-term threat recall on Day 2 for this sample, indicated by higher unpleasantness and arousal ratings, stronger cardiac deceleration (“fear bradycardia”) and increased skin conductance responses for a pair of CS+ vs. CS− that had only been presented during Day 1 acquisition. Long-term extinction recall was observed in arousal ratings and skin conductance responses as conditioned responses were reduced in a pair of CS+ vs. CS− that had undergone extinction on Day 1. For the current analyses, we estimated current source density of alpha-band activity at scalp sites consistent with visuocortical sources, to investigate (a) whether heightened visuocortical alpha suppression indexes selective visual attention to threat cues one day after conditioning, and (b) if conditioned alpha suppression is extinguished in the long term. For this purpose, we compared alpha power changes in response to previously extinguished vs. non-extinguished CS during the Day 2 recall test.

## Results

### Conditioning and extinction effects during Day 2 recall test

**Figure 1:**
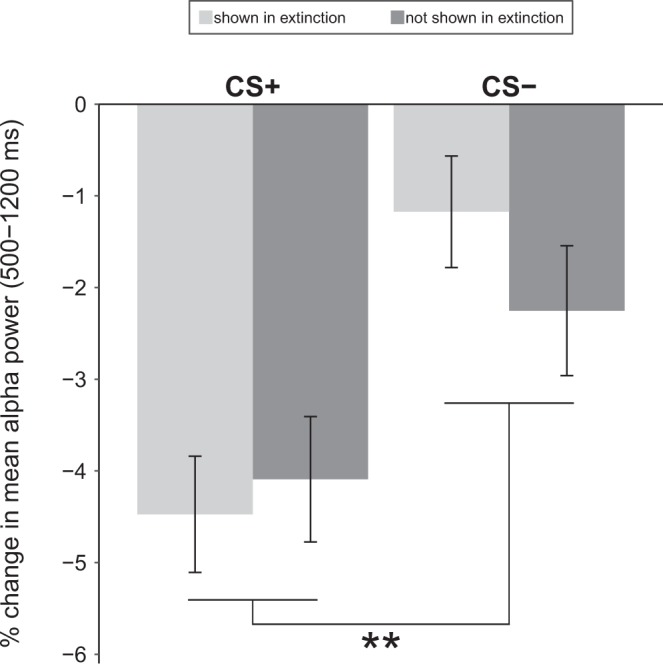
Conditioning effects on Day 2 alpha power. Mean alpha power (relative to baseline) at occipital sites in the time window of 500–1200 ms. Light bars represent CS presented during Day 1 extinction, dark bars represent CS not presented during Day 1 extinction. Error bars indicate SEM based on within-subject variance. **p < 0.01 for the main effect of Contingency.

The ANOVA showed a significant main effect of Contingency (*F*(1, 86) = 9.85, *p* = 0.002, η_p_^2^ = 0.103) as CS+ were followed by a stronger suppression of alpha power compared to CS− (also see Fig. 1). Meanwhile, the main effect Extinction (*F*(1, 86) = 0.18, *p* = 0.669, η_p_^2^ = 0.002) and the Contingency x Extinction interaction (*F*(1, 86) = 1.29, *p* = 0.259, η_p_^2^ = 0.015) were not significant.

**Figure 2:**
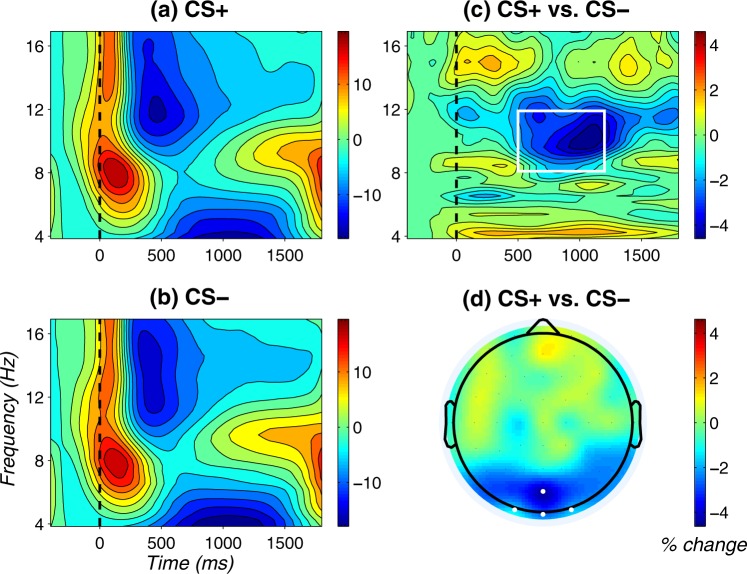
Main effect of Contingency on occipital alpha power. (**a,b**) Time-frequency plots for CS+ and CS−, respectively. Power values are % change relative to baseline (−400 to −200 ms) and averaged across Oz, POz, O1, and O2. (**c**) Time-frequency plot of the difference between CS+ and CS−, across Oz, POz, O1, and O2; the white rectangle indicates time (500 to 1200 ms) and frequency (8.1 to 11.9 Hz) windows for statistical analyses. (**d**) Topography of the difference between CS+ and CS− in the a priori defined time window (500 to 1200 ms). White dots depict the electrodes used for statistical analyses.

In line with the frequentist ANOVA, Bayesian ANOVA provided strongest evidence for a main effect of Contingency in the absence of other effects (BF_10_ = 26.0, all other models: BF_10_ < 3.3). In line with this pattern, Bayesian inclusion factors provided support for the inclusion of the main effect of Contingency (BF_Incl_ = 25.9) and against the inclusion of the main effect of Extinction (BF_Incl_ = 1 / 8.1) or the Contingency x Extinction interaction (BF_Incl_ = 1 / 4.0). Figure 2 shows time-frequency plots and topographic mapping of the Contingency effect on alpha power.

### Time window of contingency effect

**Figure 3:**
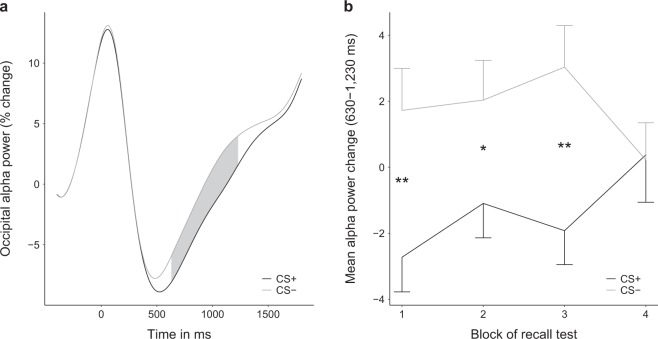
Exploratory analyses. (**a**) Time course of Contingency effect on mean occipital alpha power. Alpha power (relative to baseline) for CS+ and CS−, respectively, averaged across occipital sites. The grey-shaded area (630–1230 ms) indicates significant differences between CS+ and CS− as determined by permutation testing (p < 0.05, two-sided). (**b**) Contingency effect on mean occipital alpha power across recall test blocks. Mean alpha power (relative to baseline) for CS+ and CS−, respectively, averaged across occipital sites and from 630 to 1230 ms. *p < 0.05. **p < 0.01.

As suggested by permutation *t*-tests, viewing CS+ compared to CS− prompted significantly lower alpha power in the time window from 630 to 1230 ms post-CS (Fig. 3a), largely converging with our a priori window (500 to 1200 ms).

### Contingency effect across recall test

Exploratory analyses on occipital alpha power across recall test blocks suggested a Contingency effect: The Block x Contingency ANOVA revealed a significant main effect of Contingency (*F*(1, 86) = 9.87, *p* = 0.002, η_p_^2^ = 0.103; also see Fig. 3b) but no main or interaction effects of Block (both *p* > 0.076, η_p_^2^ < 0.027). Follow-up *t*-tests (uncorrected, two-sided) showed significant differences during the first three blocks (all *t*(86) > 2.01, *p* < 0.048) but not the last block (*t*(86) = 0.08, *p* = 0.934).

## Discussion

In the present study, we investigated the role of heightened selective attention to threat cues in long-term threat conditioning and extinction. Participants underwent a differential threat conditioning paradigm with acquisition and extinction on Day 1 and a test phase on Day 2 that allowed to assess long-term recall of extinguished and non-extinguished threat-conditioned responses. We found that, one day after threat conditioning, the CS evoked a suppression of alpha power at occipital sites between 600 and 1200 ms, which was more pronounced for threat cues (i.e., CS+) compared to safety cues (i.e., CS−). Importantly, this conditioning effect was unaffected by Day 1 extinction training.

In the present sample, we observed robust alpha power suppression at occipital electrodes that was stronger for threat-conditioned CS+ than for CS−. This is the first time that occipital alpha power suppression is shown in response to conditioned threat cues. The present effects converge with previously shown alpha suppression to naturally threatening stimuli^14,26,27^ and can be interpreted as stronger disinhibition of early visuocortical areas facilitating attentional prioritization of behaviourally more relevant (i.e., threat) cues^23,25^. They are also consistent with previous ERP research showing threat-related prioritization of visuocortical processing^7–12,30,31^. Prioritization of threat is highly important for the successful choice of adaptive responding^6,32^, given limited capacities for information processing in the brain^33,34^.

Threat-potentiated alpha suppression was found in the time window from about 600 to 1200 ms post-CS. Therefore, this alpha suppression likely indicates *sustained* attention allocation to the threat cue via recruitment of visuocortical resources^14^. This process may be initiated after initial stimulus recognition and affective stimulus categorization, which are often measured by early event-related potentials^13,35^ and may reflect subsequent in-depth analysis of threat cues as well as processes of anticipation and defensive readiness^13^. Taken together, the present results suggest that occipital alpha power changes represent a promising new marker of sustained visuocortical attention modulation in response to conditioned threat cues.

Importantly, the stronger alpha suppression in response to CS+ was evident one day after initial learning, indicating successful long-term recall of selectively increased attention allocation to threat cues. To our knowledge, this is the first study to show such long-term threat conditioning effects on a marker of visual attention, stable across sessions. The long-term stability of the present alpha suppression supports a role of selective attention modulation in the maintenance of acquired defensive responses. While there is some evidence that selective attention to threatening information contributes to the long-term maintenance of fear^36,37^, the neural underpinnings of this link are not well understood. The present study suggests a mechanism in which differential activation of visuocortical areas is a temporally stable threat-conditioned response and underlies increased attentional processing of threat cues across time. Increased attentional threat processing may consolidate fearful behaviour via increased levels of state fear^37^ and by a reduced ability to disengage from threat cues at the cost of other potentially relevant stimuli^38–40^ – e.g., concurrent safety cues or cues for successful coping. Moreover, attentional biases may interact with negative expectancy, memory, and interpretation biases related to threat^41^. Given the large number of potential mediators in fear-attention interactions, discussions regarding the role of alpha suppression in specific attentional sub-processes are speculative at this point. In order to more directly characterize attentional influences on long-term fear maintenance, future studies may probe the relationship of occipital alpha power changes to specific attention task manipulations with conditioned stimuli, and will likely include behavioural indices of attention.

In addition to long-term recall of non-extinguished conditioned threat responses (i.e., CS+N vs. CS-N), we observed comparable levels of long-term threat recall in CS that had undergone Day 1 extinction (i.e., CS+E vs. CS-E), possibly due to non-successful Day 1 extinction or spontaneous recovery (“return of fear”, also see supplement)^1,42^. This pattern of long-term extinction resistance (i.e., threat recall in CS previously undergoing extinction) was corroborated by Bayesian analyses, which favoured models excluding the extinction factor. Note that the finding of Day 2 alpha suppression in previously extinguished CS mirrors the pattern of cardiac deceleration in the present sample^29^ as well as in previous studies^43,44^. Threat-evoked cardiac deceleration has been considered a measure of attentional processes, namely orienting in the face of imminent harm^45–47^. This suggests that attention-related processes in general, and occipital alpha suppression in particular, could be more extinction-resistant in the long term than other conditioned responses and may occur in a better-safe-than-sorry fashion^48^. In other words, the cost of increased attentional engagement to invalid threat cues (i.e., false alarms) may be judged as significantly smaller than the cost for overlooking valid threat cues – even after repeated omission of harmful events.

As discussed earlier, alpha-related attentional processes may be crucial in the long-term maintenance of fears. The extinction resistance of alpha changes observed in the present study suggests that former threat cues continue drawing on attentional resources even in the absence of contingent reinforcement by harmful outcomes, i.e., even when cues are not followed by negative consequences anymore after initial learning. Moreover, occipital alpha suppression may prove useful for investigating the influence of threat-focused attention and attentional biases on the effectiveness of exposure therapy^49,50^.

The current study had several limitations. First, using the current paradigm, it cannot be ruled out that CS+ evoked stronger alpha suppression than CS− due to partial reinforcement and changing contingencies across phases, making predictions of US occurrence more difficult in CS+ vs. CS− and motivating participants to process CS+ more thoroughly. This, however, is unlikely to explain the present effects given that threat-depicting pictures, with no learning history, also evoked stronger alpha suppression in previous studies^14,26,27,51^. Second, we only used male participants in order to investigate conditioning and extinction without the influence of known sex differences^52^. Because there may be gender differences in visuocortical threat processing^53,54^, the present results should be replicated in women. Third, we collected no EEG during acquisition and only a limited amount of trials during extinction on Day 1 as the current study was primarily designed to test long-term conditioning effects. Future studies may collect larger amounts of trials during initial acquisition and extinction, thereby providing a more complete picture of the learning dynamics as reflected in alpha power fluctuations to threat. Nevertheless, the current experimental design allows a clear interpretation of the present Day 2 alpha power suppression as a function of Day 1 learning.

In the present study, we could show that conditioned visual threat cues evoke enhanced alpha suppression at occipital sites. Moreover, we showed for the first time that increased attention allocation to conditioned threat cues via sustained recruitment of early visuocortical areas is temporally sustained over a 24 h period, stable, and resistant to extinction. Future studies may use occipital alpha power to examine mechanisms of visual attention in the development, maintenance, and extinction of fears.

## Methods

### Sample

We reanalysed data of *N* = 87 healthy, male participants (mean age: 23.7, *SD*: 3.85, range: 18–34; data collected from *N = *93; *n* = 6 excluded both from previous and current analyses due to insufficient EEG quality), described in more detail elsewhere^29^. For the purpose of the previously reported genetic analyses, participants had been preselected based on the dopaminergic *COMT* Val158Met polymorphism yielding comparable group sizes for each genotype (Val/Val: *n* = 30, Val/Met: *n* = 29, Met/Met: *n* = 28). Participation was compensated with 65 € for two experimental sessions on subsequent days (Day 1: 9 am – 2 pm; Day 2: 3 pm – 5 pm). The study was conducted in accordance to the Declaration of Helsinki and was approved by the ethics committee of the German Psychological Association (DGPs). Informed consent was obtained from all participants at the beginning of the experiment.

### Experimental design

#### Conditioning and extinction paradigm

**Figure 4:**
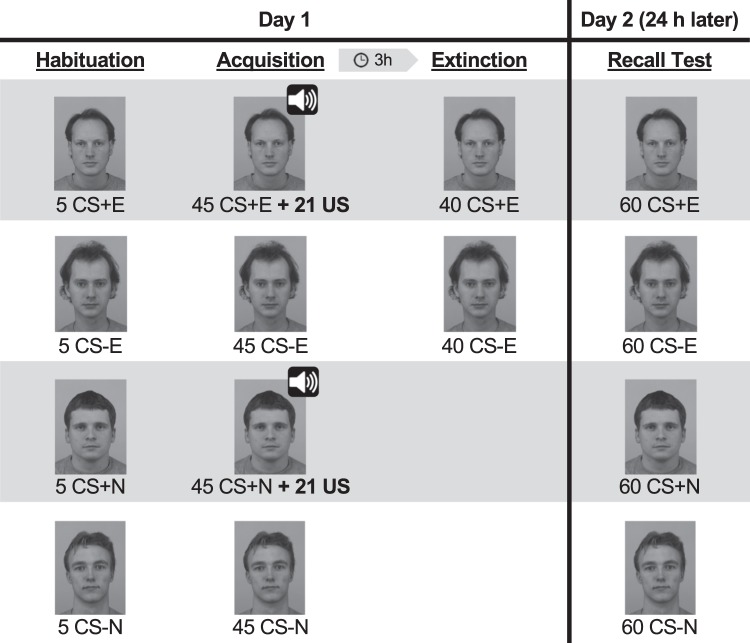
Conditioning and extinction paradigm. Face stimuli and number of presentations in the two-day differential threat conditioning and extinction paradigm. The US was only presented during acquisition, indicated by the speaker symbol. Assignment of different faces to CS type was permutated across participants. CS+ E = extinguished CS+, CS+ N = non-extinguished CS+, CS-E = CS− presented during extinction phase, CS−N = CS− not presented during extinction phase. Stimuli were presented in colour. KDEF stimuli IDs from top to bottom: AM10NES, AM13NES, AM31NES, BM08NES.

We employed a two-day differential threat conditioning and extinction paradigm^29,55^ (also see Fig. 4). Briefly, on Day 1, participants underwent CS habituation (5 presentations per CS), followed by an acquisition phase. Each of two CS (CS+ E, CS+ N) was paired with a US in 21 out of 45 trials (46.6%), the other two CS (CS-E, CS-N) were never paired with the US (also 45 trials per CS). After the acquisition phase, electrodes were removed, participants received a light standardized breakfast, completed a series of personality questionnaires, and electrodes were attached for the extinction phase. The extinction phase started exactly three hours after the end of acquisition and consisted of 40 CS+ E and CS-E presentations (‘E’ standing for *presented during extinction phase)*. CS+ N, CS-N, and US were not presented during the extinction phase (‘N’ standing for *not presented during extinction phase)*. Approximately 24 h after the extinction, participants returned for a Day 2 recall test which included 60 trials of each CS. No US were presented on Day 2. Participants were asked to rate the CS regarding valence and arousal on multiple occasions (data reported in^29^).

#### Stimuli and trial structure

We used four different male faces with neutral expression from the Karolinska Directed Emotional Faces set^56^ as CS (pictures: AM10NES, AM13NES, AM31NES, BM08NES; also see Fig. 4). Assignment of face stimuli to the different CS types was permutated and balanced across participants. The US was a 1 s white noise burst at 95 dB(A) delivered by a room speaker as we had previously shown that noise bursts are particularly well suited for threat conditioning with many trials^44^. In every trial, a fixation cross (1 s duration) was presented before participants saw the CS for 4 s. In paired trials the CS co-terminated with the US for 1 s. A black screen (jittered duration, 6–8 s) was presented between trials.

### EEG recording and preprocessing

64-channel EEG was recorded with a QuickAmp72 amplifier and actiCAP active electrodes (Brain Products, Gilching, Germany) at 1000 Hz, with a 200 Hz online lowpass-filter and referenced against the average. EEG processing was performed in BrainVision Analyzer 2 (Brain Products, Gilching, Germany). The EEG was downsampled to 500 Hz, highpass-filtered (−3 dB at 0.1 Hz, 24 dB/oct., zero-phase IIR Butterworth filter) and notch-filtered (50 Hz, 5 Hz bandwidth, 16th order). In order to increase signal stationarity required for ICA, large EEG artefacts were removed manually, and the signal was 0.5 Hz highpass-filtered. Extended Infomax ICA^57^ was applied to the continuous data. Critical independent components reflective of horizontal and vertical eye movements, blinks, and cardiac artefacts were identified and removed by an experienced rater. Resulting ICA weight matrices were used to transform the 0.1 Hz filtered EEG data into independent components, remove the critical components, and transform the remaining components back into EEG data^58^. Segments with remaining artefacts (e.g., steep transients, slow drifts) were removed manually resulting in a comparable number of included trials for CS+E (*M* = 43.1, *SD* = 8.7, range = 16–57), CS+N (*M* = 42.1, *SD* = 8.6, range = 19–54), CS-E (*M* = 43.7, *SD* = 7.9, range = 22–57), and CS-N (*M* = 43.2, *SD* = 8.5, range = 20–57). Channels with excessive amounts of artefacts (mainly voltage drifts/spikes and remaining line noise caused by poor electrode contact) were interpolated (spline interpolation).

Continuous EEG data were lowpass-filtered (30 Hz, filter specifications identical to highpass filter). After correcting marker latencies for monitor delay (33 ms), data were segmented relative to CS onset (−600 to 2000 ms).

To estimate current source density and improve the spatial specificity of the voltage map, the surface Laplacian was computed^59^ as implemented in BrainVision Analyzer 2 (spline order: 4, Legendre polynomial: 10, lambda: 1e-5). In order to facilitate comparison with previous studies, we also analysed the average-referenced scalp data (i.e., without Laplacian transformation). The results converged with the present results in the surface Laplacian data and are provided as supplementary material.

### Wavelet analyses

Wavelet analyses were conducted in MATLAB 2013a (MathWorks, Natick, MA, USA) using established custom scripts^60^. First, EEG segments in the time domain were baseline-corrected (subtraction of the mean from −600 to −500 ms) and tapered with a cosine square window (20 samples rise/fall). Complex Morlet wavelets were applied with variable bandwidths (Morlet parameter = f/σ_f_ = 12) to compute power for frequency bands from 3.8 to 30.4 Hz in linear steps of 0.38 Hz. Power in each frequency band was baseline-corrected by dividing the signal by the mean amplitude between −400 ms and −200 ms relative to CS onset. Resulting values were rescaled to percentage change relative to baseline. Mean power of all discrete frequencies from 8.1 Hz to 11.9 Hz^14,15^ was used for statistical analyses on alpha.

### Statistical analyses

Statistical analyses were performed with R^61^ in the RStudio environment^62^. In order to assess the influence of Day 1 learning on occipital alpha during Day 2, we computed mean alpha power across Oz and adjacent electrodes (Oz, POz, O1, and O2) in the time window of 500–1200 ms^27,63^ after the CS. The resulting values were entered into an ANOVA with the within-subject factors Contingency (CS+ vs. CS−) and Extinction (extinguished vs. non-extinguished) using the *aov* function in R. Type I error level was set to α = 0.05. Distribution of mean alpha power across participants was sufficiently close to a normal distribution as indicated by low values of skewness and kurtosis for all CS types (|skewness| ≤ 0.46, |kurtosis| ≤ 1.12)^64^. In addition to null-hypothesis testing, we conducted Bayesian analyses as implemented in the *anovaBF* function in the BayesFactor package^65^ for R. We computed Bayes factors (100,000 iterations) for four different models (main effect Contingency only, main effect Extinction only, additive model of Contingency + Extinction main effects, complete model including both main effects and the interaction term^66^) compared to the null model (BF_10_). All models had equal prior probabilities and included a random effect term to account for between-subject variance. In a second step, we computed Bayesian inclusion factors (BF_Incl_) for each effect (i.e., Contingency, Extinction, and Contingency x Extinction) that indicate whether models including this effect are more likely to explain the data than matched models without this effect. More precisely, all models containing the effect of interest – but no higher-order interactions of this effect – were compared to matched models stripped of the effect (BAWS factor suggested by Sebastiaan Mathôt; also implemented in JASP 0.9^67^). As an example, for the Contingency effect, the models *Contingency only* and *Contingency* + *Extinction* were compared to the null model and the *Extinction only* model.

### Exploratory analyses

After using a predefined time window (500 to 1200 ms) for hypothesis testing, we conducted follow-up analyses on the exact time window of the Contingency effect based on the present data. We used permutation-controlled *t*-tests (adapted from the *t*_max_ approach from Blair and Karniski^68^), comparing alpha power (averaged across Oz, POz, O1, O2) at each time sample between both CS+ and both CS− (i.e., Contingency contrast). First, we randomly permutated the CS+ and CS− condition in each participant 1,000 times and computed *t*-values for each of the 1,300 time samples. Then, the tails of each permutation’s *t*-value distribution were determined and stored in a *t*_min_ and *t*_max_ distribution, respectively, each having 1,000 values corresponding to the 1,000 permutations. Finally, 2.5^th^ and 97.5^th^ percentiles from the resulting distribution of *t*_min_ and *t*_max_ values were used as critical *t*-values to compare empirical *t*-values against (i.e., α = 0.05, two-sided; *t*_crit_: CS+ < CS−: −2.59; CS+ > CS−: 2.64).

Using the time window as determined by permutation tests (630–1230 ms), we conducted an analysis of the Contingency effect’s stability across the recall test. For this purpose, we separated all available trials into 4 equally-sized blocks for each CS, respectively. Mean occipital alpha power change for CS+ and CS− was computed for each block. We then conducted an exploratory Block (1 vs. 2 vs. 3 vs. 4) x Contingency (CS+ vs. CS−) ANOVA (*ezANOVA* function^69^; type III SS; Greenhouse-Geisser correction) and computed four separate *t*-tests comparing CS+ and CS− in each block (all α = 0.05).

## Supplementary information

Supplementary Analyses

## Data Availability

Data and R scripts for statistical analyses as well as preprocessed single-trial EEG data of the current study are available in the Open Science Framework (OSF) repository, osf.io/bfqjc.
