# Supplementary material for: Extinction-resistant attention to long-term conditioned threat is indexed by selective visuocortical alpha suppression in humans
Source: Sci Rep. 2019 Nov 1;9:15809. doi: 10.1038/s41598-019-52315-1 (PMC6825167; doi:10.1038/s41598-019-52315-1)
Supplement: Supplementary file 1 — Supplementary Analyses [file 41598_2019_52315_MOESM1_ESM.pdf]

Extinction-resistant attention to long-term conditioned threat is indexed by selective visuocortical alpha suppression in humans

Christian Panitz, Andreas Keil, & Erik M. Mueller

Supplementary Material

Occipital alpha power during Day 2 recall test – average-referenced scalp EEG

In order to facilitate comparison with de Cesarei & Codispoti (2011) and Vagnoni et al. (2015), we conducted analyses on posterior alpha power using average-referenced EEG at all available parieto-occipital sites (here: P7, P5, P3, P1, Pz, P2, P4, P6, P8, PO9, PO7, PO3, POz, PO4, PO8, PO10, O1, Oz, O2). All other parameters (e.g., time window, wavelet parameters) are identical to the main analysis of the present manuscript.

In line with the main analyses on CSD data, the ANOVA showed a significantly stronger alpha suppression to CS+ vs CS- (main effect of Contingency: $F(1, 86) = 15.3$, $p < .001$, $\eta_{p2} = .151$; Supplementary Figure 1). The main effect Extinction ($F(1, 86) = 0.88$, $p = .350$, $\eta_{p2} = .010$) and the Contingency x Extinction interaction ($F(1, 86) = 0.95$, $p = .333$, $\eta_{p2} = .011$) were not significant.

In line with the frequentist ANOVA, Bayesian ANOVA provided strongest evidence for a main effect of Contingency in the absence of other effects ($BF_{10} = 90.1$, all other models: $BF_{10} < 16.4$). In line with this pattern, Bayesian inclusion factors provided support for the inclusion of the main effect of Contingency ($BF_{Incl} = 90.6$) and against the inclusion of the main effect of Extinction ($BF_{Incl} = 1 / 5.5$) or the Contingency x Extinction interaction ($BF_{Incl} = 1 / 3.9$). Supplementary Figure 2 shows time-frequency plots and topographic mapping of the Contingency effect on alpha power on average-referenced EEG.

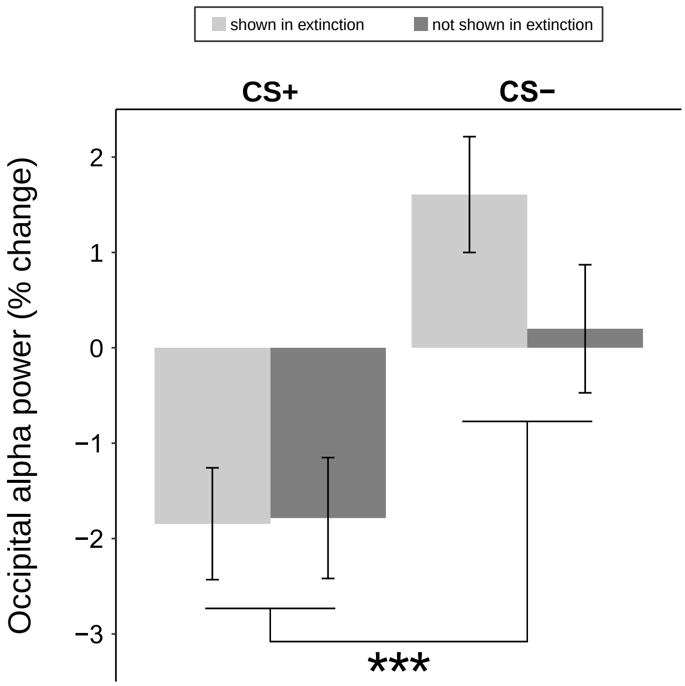

Supplementary Figure 1. **Conditioning effects on Day 2 alpha power.** Mean alpha power (relative to baseline) at P-, PO-, and O-electrodes in the time window of 500 – 1200 ms. Light bars represent CS presented during Day 1 extinction, dark bars represent CS not presented during Day 1 extinction. Error bars indicate SEM based on within-subject variance. ***p < .001 for the main effect of Contingency.

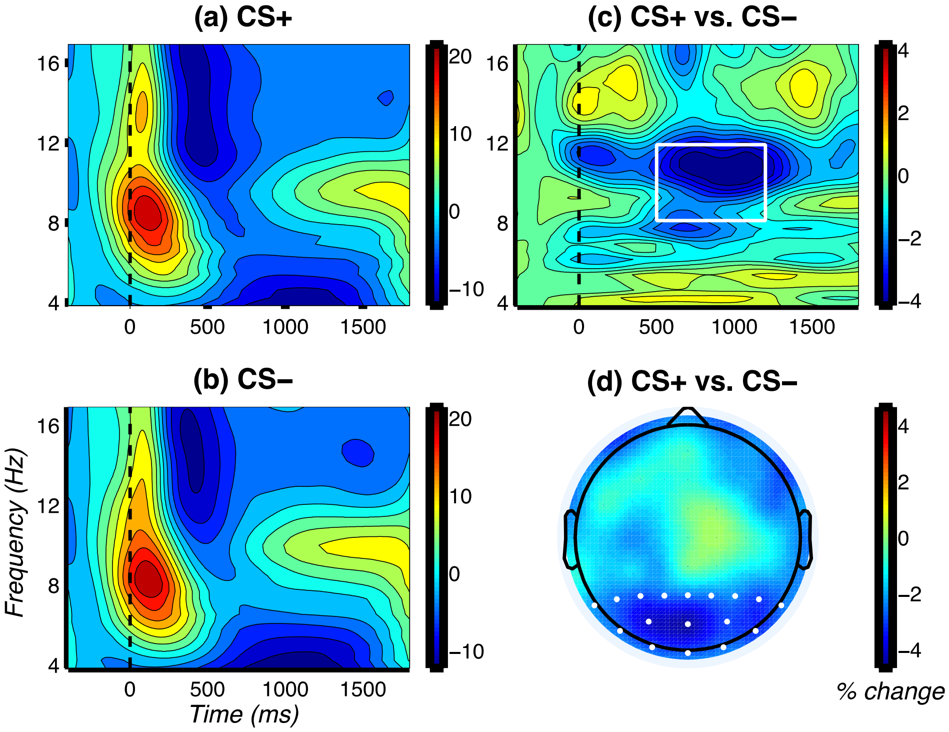

Supplementary Figure 2. **Main effect of Contingency on occipital alpha power**. (a) and (b) Time-frequency plots for CS+ and CS-, respectively. Power values are % change relative to baseline (-400 to -200 ms) and averaged across all P-, PO, and O-elecrodes. (c) Time-frequency plot of the difference between CS+ and CS-, across all P-, PO-, and O-electrodes; the white rectangle indicates time (500 to 1200 ms) and frequency (8.1 to 11.9 Hz) windows for statistical analyses. (d) Topography of the difference between CS+ and CS- in the a priori defined time window (500 to 1200 ms). White dots depict the electrodes used for statistical analyses.

Occipital alpha power during Day 1 extinction

All signal preprocessing was identical to the Day 2 recall test data reported in the main manuscript (CSD data; electrodes: Oz, O1, O2, POz; time window: 500-1200 ms). For statistical analysis, we computed alpha power for two blocks (i.e., the first and second half of extinction trials, respectively). While the descriptive pattern suggested within-session extinction of conditioned alpha power suppression, the Block (Block 1 vs Block 2) x Contingency (CS+E vs. CS-E) ANOVA revealed no significant effects (all $p > .070$, $\eta_{p2} < .038$; Supplementary Figure 3). Given the low trial number (20 trials per Block/CS combination), and the associated reduced signal-to-noise ratio of the spectral estimates, results should be interpreted with caution.

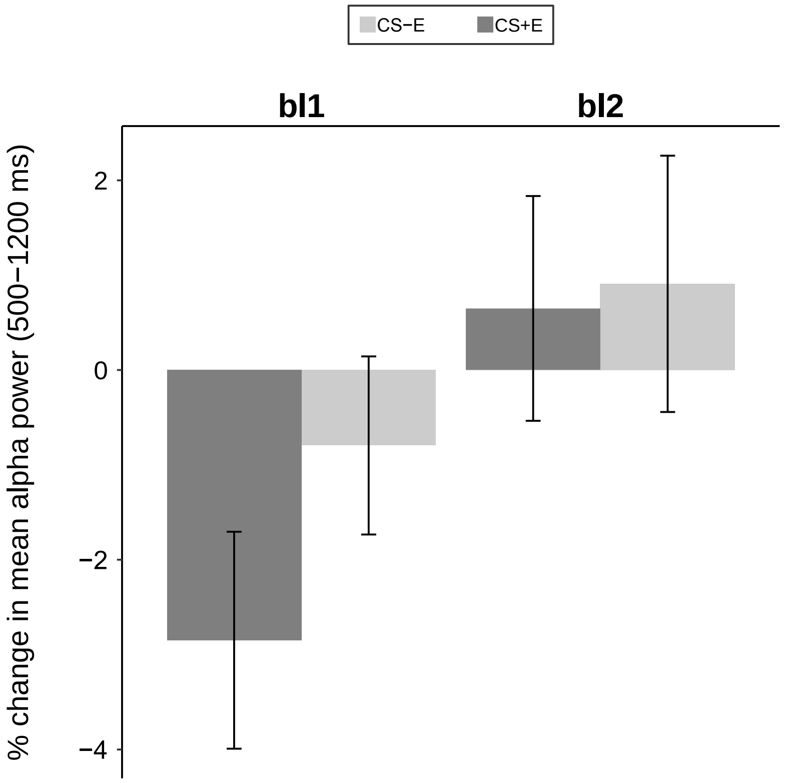

Supplementary Figure 3. **Day 1 extinction alpha power.** Mean alpha power (relative to baseline) at Oz, O1, O2, POz in the time window of 500 – 1200 ms. Error bars indicate SEM based on within-subject variance.
